# Maternal awareness, acceptability and willingness towards respiratory syncytial virus (RSV) vaccination during pregnancy in Ireland

**DOI:** 10.1002/iid3.1257

**Published:** 2024-04-25

**Authors:** Siobhan McCormack, Claire Thompson, Miriam Nolan, Mendinaro Imcha, Anne Dee, Jean Saunders, Roy K Philip

**Affiliations:** ^1^ Division of Neonatology, Department of Paediatrics University Maternity Hospital Limerick Limerick Ireland; ^2^ Department of Midwifery University Maternity Hospital Limerick Limerick Ireland; ^3^ Department of Obstetrics and Gynaecology University Maternity Hospital Limerick Limerick Ireland; ^4^ Department of Public Health Medicine Health Service Executive Limerick Ireland; ^5^ Claddagh Statistical Consulting Services (CSCS), Shannon & Limerick Limerick Ireland; ^6^ University of Limerick School of Medicine Limerick Ireland

**Keywords:** life‐course immunization, maternal immunization, maternal vaccination, pregnancy, respiratory syncytial virus, vaccination, vaccine confidence

## Abstract

**Background:**

Respiratory syncytial virus (RSV) is the world's leading cause of viral acute lower respiratory infections (ALRI) in infants. WHO has identified maternal RSV vaccination a priority and candidate vaccines are in development; however, vaccine hesitancy remains an impediment to successful implementation of maternal immunization. This study, the largest antenatal survey conducted to‐date, aimed to examine maternal RSV awareness, likely acceptance of RSV vaccination in pregnancy, and attitudes to maternal vaccination.

**Methods:**

Pregnant women of all gestations attending antenatal clinic of a university maternity hospital in Ireland were invited to participate. An information leaflet provided, consent obtained, and survey administered examining RSV awareness, willingness to avail of antenatal RSV vaccination, factors influencing acceptability and preferred sources of assistance. Research Ethics Committee (REC) approval obtained, and general data protection regulation (GDPR) guidelines followed.

**Results:**

528 women completed the survey. A large proportion (75.6%) had never heard of RSV, yet 48.5% would still avail of a vaccine, 45.8% were undecided and only 5.3% would not. The main factor making vaccination acceptable to women (76.4%) was that it protects their infant from illness (*p* < .001, CV 0.336 for association with acceptance) and general practitioner (GP) was the preferred guidance source in decision‐making (57.7%).

**Conclusions:**

Despite low levels of maternal awareness of RSV, pregnant women in Ireland are open to availing of antenatal vaccination. Maternal immunization strategies need to focus on infant's protection from RSV‐associated ALRI along with vaccine safety, and build on an interdisciplinary collaboration of maternal, neonatal, primary care and public health services.

## Graphical abstract

A study of maternal awareness, acceptability and willingness towards antenatal respiratory syncytial virus (RSV) vaccination in Ireland showed low levels of awareness of RSV but an openness to this approach to protect infants. Collaborative working will be required to raise awareness and facilitate maternal vaccine uptake, as part of a life‐course immunization strategy.

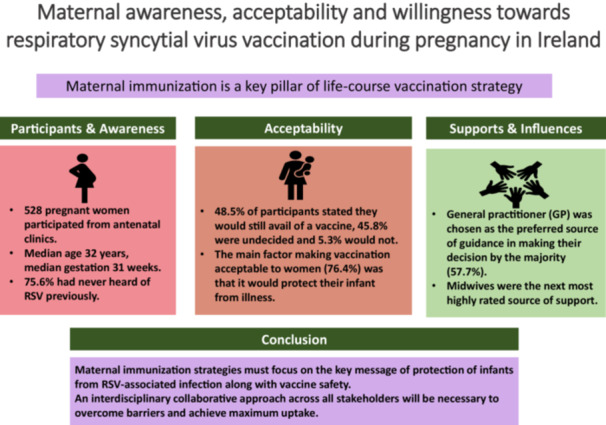

## 1 INTRODUCTION

Pregnancy and neonatal period poses an increased vulnerability to infection secondary to a relative immune suppression.^1^ Infections during early infancy can potentially lead to high rates of morbidity, invasive disease, and mortality.^2^ Maternal immunization strategies are being increasingly acknowledged and explored for their potential to influence the health of both women and their infants, as part of a life‐course immunization (LCI) approach.^3^ Maternal immunization is recognized as a key primary preventative approach that could directly benefit two generations of the population through a single intervention.^4^ Maternal immunization, resulting in passive immunity through transfer of IgG antibodies has been shown to be effective for the prevention of pertussis,^5^ influenza^6^ tetanus,^7^ and more lately SARS Cov‐2^8^ in infants and data supports that similar strategies could be effective for the prevention of other infections, including Respiratory Syncytial Virus (RSV).^9^ Programs for the prevention of pertussis and influenza through maternal immunization are in place in many countries, however, they are associated with varying, often suboptimal, acceptance rates.^10^ This sits against a backdrop of worldwide vaccine uptake being below recommended levels for all age groups at a time where the World Health Organisation (WHO) has designated vaccine hesitancy as a substantial threat to global health.^11^

RSV has been identified as a high priority for vaccine development by the WHO^12^ and this study is important in our understanding of maternal awareness and knowledge of RSV, along with acceptability for such a vaccination and attitudes and influences towards antenatal vaccination generally, to maximize uptake. This has seldom been examined before in large numbers of pregnant women, with only two reported studies involving views on antenatal RSV vaccination—one analyzing 495 women from a largely Australian and southeast Asian population^13^ and the other among 314 pregnant women in the UK.^14^

### 1.1 Burden of respiratory syncytial virus infections

RSV is the leading cause of acute lower respiratory infections (ALRI), encompassing both bronchiolitis and pneumonia, and leads to one of the commonest reasons for hospitalization among infants.^15^ RSV causes up to 80% of cases of bronchiolitis and 20% of pneumonia among infants and young children.^15^ There were over 3600 cases of RSV reported in Ireland in winter 2018/2019, which was the highest number of notifications recorded before COVID‐19 pandemic, since RSV became a notifiable illness in Ireland in 2012.^16^ While during the COVID‐19 pandemic and lockdowns RSV infection declined drastically, there has been a resurgence with even higher RSV rates once the stringent public health mitigation measures against COVID‐19 were discontinued.^17^, ^18^ Globally, in 2019, 33 million episodes of RSV‐associated ALRI, 3.6 million RSV‐associated hospital admissions, 26 300 RSV‐associated ALRI related in‐hospital deaths, and 101,400 RSV‐attributable overall deaths occurred in infants and children aged 0–60 months, with almost half of these occurring in infants under 6 months.^19^ RSV‐associated ALRI is established as the most common cause of death due to ALRI worldwide for infants in the Global Burden of Disease Study.^20^ Research suggests that the vast majority of young children would have experienced at least one RSV infection by 2 years of age.^21^ In temperate climates, RSV incidence shows a clear seasonality with onset in autumn or early winter, peaks from mid‐December to February, posing significant pressure on health services, and near cessation of cases by early spring.^22^ Severe RSV‐associated ALRI often demands admission to pediatric high dependency units (PHDU) or pediatric intensive care units (PICU), and increases the pediatric critical care transport demands during peak RSV seasons.^23^ RSV‐associated ALRI has also been associated with increased rates of subsequent development of early recurrent wheeze, asthma, and possibly allergic senitization later in childhood.^24^ All of these potential associations carry substantial healthcare burden and cost, along with negative influence on quality of life.^25^

### 1.2 RSV prevention

Infants are most vulnerable to infection from RSV in their first weeks to months of life and as such, active immunization of infants against RSV would be extremely challenging to achieve immunity when it is most needed.^26^ Currently, passive immunity is conferred to selected list of high‐risk infants through a monthly intramuscular injection during RSV season with palivizumab, the only commercially available short‐acting monoclonal antibody (mAb), which targets the RSV fusion (F) protein, and reduces the risk of hospitalization and severe infection with RSV‐associated ALRI. High‐risk infants qualifying for palivizumab include preterm infants <29 weeks gestation, chronic lung disease of prematurity (CLD), hemodynamically significant congenital heart disease, cystic fibrosis, Down syndrome, neuromuscular disorders and immunocompromised,^27^, ^28^ and the product as well as monthly administration are expensive.^29^ Several candidate vaccines for pregnant women to protect their newborn infants against RSV infection are currently at various stages of development, and the first Phase 3 clinical trial was reported in early 2020.^30^ This study involving a recombinant RSV F protein nanoparticle vaccine in a trial of 4636 pregnant women did not reach its primary endpoint criteria but demonstrated 39.4% (95% confidence interval [CI] 5.3%–61.2%) efficacy against medically significant RSV‐associated ALRI in the first 90 days and findings on other endpoints suggest potential benefits. In terms of safety, the adverse events in those who received the vaccine was similar to the placebo.^30^ Another maternal RSV vaccine Phase 3 trial was found to be 82% effective in preventing severe medically attended ALRI due to RSV in newborns up to 90 days after birth, and 69% effective for the first 6 months after birth.^31^ Designated RSVpreF, this vaccine blocks RSV pre‐fusion F protein, preventing the virus from entering human cells.^31^ On the other hand, one Phase 3 clinical trial of candidate RSV maternal vaccine was discontinued in February 2022 due to safety concerns.^32^ A number of other candidate vaccines are also currently at various stages of development.^33^, ^34^, ^35^ Since both maternal vaccination, and the recently published longer‐acting mAb (Nirsevimab) appears to render protection against RSV‐associated ALRI among young infants, either of them or a combination could offer reduction of RSV disease burden, and potentially reduce mortality in low and middle‐income countries (LMIC).^36^, ^37^ With multiple maternal candidate vaccines progressing through the developmental process, it is prudent to assess the awareness and knowledge of RSV infection of infants among pregnant women, and acceptability of vaccination when ready for approval for maternal use, and pre‐empt the barriers to successful implementation of a maternal immunization strategy to protect infants against RSV‐associated ALRI.

#### 1.2.1 AIMS

The primary aim of this study was to evaluate maternal awareness and knowledge of RSV infection and to assess likely acceptance if an approved, licenced vaccine for use in pregnancy is made available. In addition, the secondary objectives of the study aimed to establish attitudes to antenatal vaccination, factors influencing acceptance and the desired sources of information.

## 2 METHODS

### 2.1 Study participants

Pregnant women of all gestations were invited to participate in this paper‐based survey. Ethical approval was granted by the University of Limerick Hospitals Group research ethics committee (REC Ref:101/18). No incentives were offered for participation in the study. An information leaflet on RSV infections was provided. Consent for survey participation and the additional option of the use of relevant anonymized antenatal data was obtained and General data protection regulation (GDPR) guidelines were followed. Study design proposed a convenience sample size of 10% the annual number of pregnant women attending the service (10% of 4500 women, i.e., 450 women) for enrollment. To compensate for subset analysis sufficiency, survey completion inaccuracy/errors and to reflect on the standard deviation of the convenience sample, 1%–2% of additional survey enrollment was recommended. The survey was not stratified based on socioeconomic status/education, and being undertaken in a publicly funded antenatal clinic, predominant patient population may not have private health insurance.

### 2.2 Survey administration

The survey was conducted during the routine and high‐risk public antenatal clinic visits using a convenience sample approach. Surveys were carried out from December 2018 to April 2019 at a University maternity hospital in the Mid‐West of Ireland. A member of the research team was available to answer any questions in relation to the survey or participation. Women were invited to take part in the survey once (irrespective of gestational age) throughout the study period.

### 2.3 Survey questions

The survey questions, along with the patient‐facing information leaflet materials provided, are contained in Supporting Information: S1 and S2. No validated maternal vaccination survey instrument for the population of pregnant women is available. The survey instrument in this study was designed to answer the research questions, addressing knowledge of RSV and acceptability of antenatal RSV vaccination. In this study, pregnant women were asked if they would avail of a vaccine rather than get or purchase, reflecting the Irish context where vaccines advised in pregnancy as per national immunization policy are provided free of charge to those who wish to avail of them.

### 2.4 Data collection and statistical analysis

Anonymised data were collected from the paper surveys using Microsoft Excel and relevant antenatal information was obtained from the computerized hospital inpatient management systems (iPMS) for women who had given additional consent for this, as outlined in the consent form. Health Service Executive (HSE) guidelines on GDPR was followed for the collection, recording and analysis of deidentified and fully anonymised data.^38^

Statistical analyses were conducted using SPSS version 25 (IBM). Statistical analysis on availing of RSV vaccination if offered was tested for differences in age, gestational age, gravidity, and opinion about vaccines using the nonparametric Kruskal–Wallis (KW) tests for the scalar measurements and chi‐squared tests for the categorical variables. KW test was preferred in the context of comparing two or more independent sample parameters. KW test does not assume normal distribution of residuals and a significant KW test indicates that at least one sample characteristic stochastically dominates another. P‐values < 0.05 were considered statistically significant.

## 3 RESULTS

### 3.1 Demographics

652 women were invited to take part in the study with 528 women completing the questionnaire, giving with a response rate of 81%. In 42 women their age and gravidity were not obtained due to lack of consent, or the information was not disclosed, in the remaining 486 women the median age was 32 years (interquartile range 28–36).

Gestation ranged from 8 to 40 weeks with a median of 31 weeks (interquartile range 26–36). 50.2% of women were in their first pregnancy, 24.5% in their second and the remaining 25.3% in their third or subsequent pregnancies with overall median gravidity of 1 (interquartile range 1–3) (Table 1).

**Table 1:** Participant demographics.

Parameter	Median	Interquartile range (IQR)
Age	32 years	IQR (28–36)
Gestation	31 weeks	IQR (26–36)
Gravidity	1	IQR (1–3)

### 3.2 Awareness of RSV

**Figure 1:**
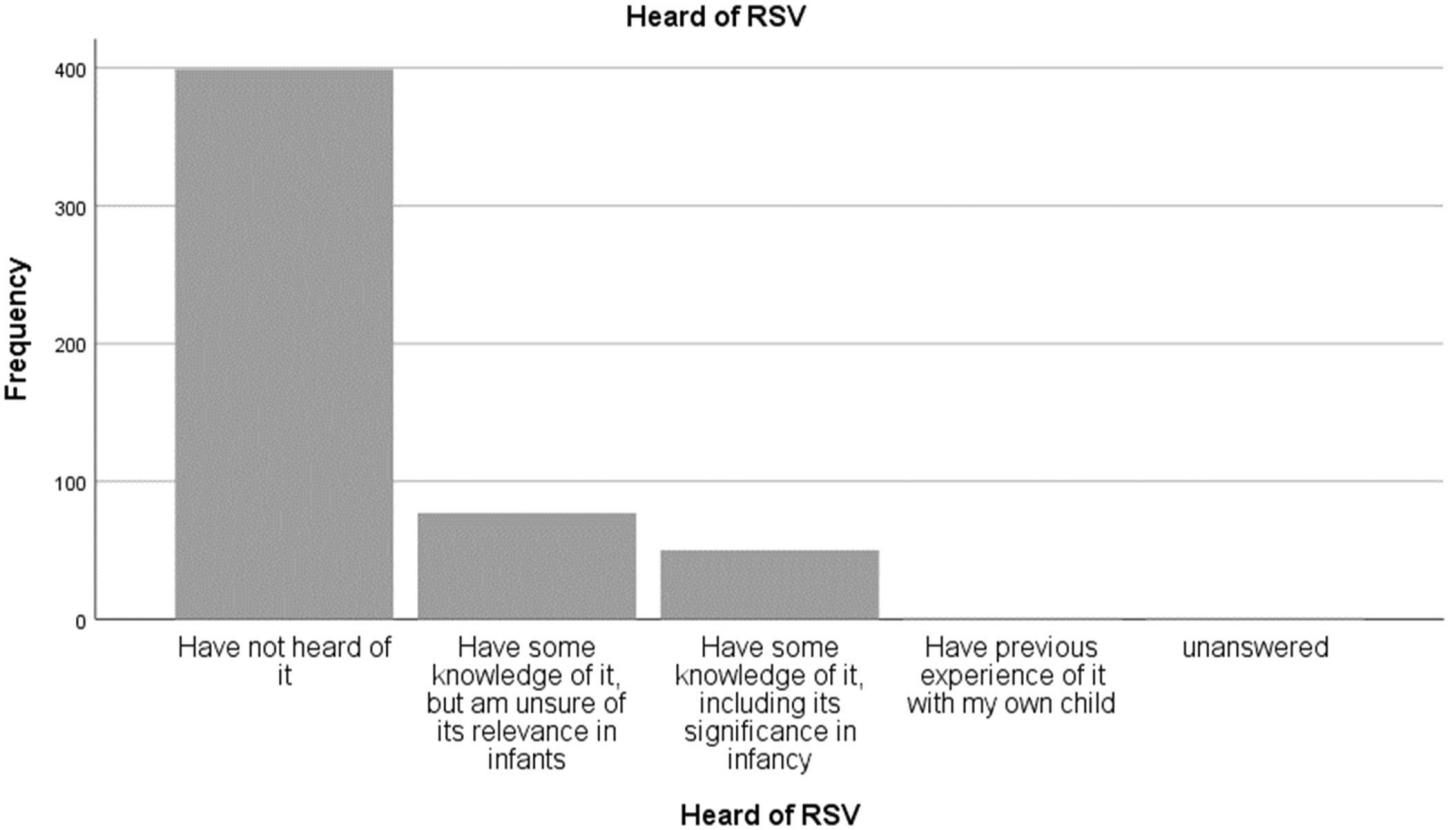
Pregnant women's awareness of respiratory syncytial virus (RSV).

When asked whether they had heard of RSV, 399 (75.6%) respondents had not heard of it, 77 (14.6%) had some knowledge of it but were unsure of its significance, 50 (9.5%) had some knowledge of it including its significance in infants and only 1 (0.2%) indicated they had previous experience of it in their own child (Figure 1).

### 3.3 Acceptability of antenatal vaccination against RSV

Whether they would avail of an antenatal RSV vaccine if available, 256 (48.5%) reported yes. 242 (45.8%) reported they did not know and 28 (5.3%) opted for no (Table 2).

**Table 2:** Responses on acceptability of RSV vaccination.

Option selected	Response *n* (%)
Yes	256 (48.5)
Don't Know	242 (45.8)
No	28 (5.3)

Using Kruskal–Wallis analysis, the distribution of age was not significantly different between the availing of RSV vaccine groups, *p* = .644, nor was the distribution of gestation (*p* = .324), or gravidity (*p* = .201).

### 3.4 Attitudes towards vaccination

When asked what makes vaccination acceptable, respondents were invited to indicate all the statements that applied to them and many therefore gave more than one answer: Table 3.

**Table 3:** Attitudes towards vaccination.

Statements selected	*N* (%)
‘I feel recommended vaccines will protect my baby from illness’.	356 (67.4)
‘I feel confident in recommended vaccines’.	258 (48.9)
‘I feel the vaccines could harm my baby’.	57 (10.8)
‘I don't think my baby is at risk of infection’.	22 (4.2)
‘I feel the vaccines could harm me’.	19 (3.6)
‘I have no confidence in vaccines’.	15 (2.8)

### 3.5 Factors affecting acceptance of RSV vaccination

Some of the responses about vaccines were tested against their willingness to avail of antenatal RSV vaccine, where the numbers giving that answer were sufficient for analysis.

Analyzing vaccine acceptability, 62% of those who felt confident in vaccines and 56% of those who feel vaccines will protect their baby from illness would avail of RSV vaccination, factors which increase acceptability from an overall 48.5%.

**Figure 2:**
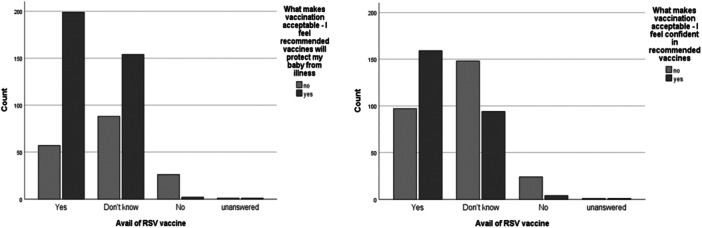
Factors significantly affecting antenatal vaccination acceptance.

Regarding the answers “I feel confident in recommended vaccines” and “I feel recommended vaccines will protect my baby from illness,” when tested against availing of RSV vaccine (Figure 2) there was a significant association with more saying yes to each statement and that they would avail of RSV vaccination than expected by chance. For “I feel confident in recommended vaccines” (*p* < .001, cv = 0.279), and, for “I feel recommended vaccines will protect my baby from illness” (*p* < .001, cv = 0.339), both offering a moderate association.

Finally, their awareness of the condition and their likelihood of availing of the vaccine was tested for association. Those with knowledge or prior experience of RSV were not significantly more likely to avail of the vaccine but this did have borderline significance and may have been significant if the sample size and power of the study were larger, *p* = .072, cv = 0.112.

### 3.6 Decision‐making influences in vaccination acceptance

When asked what their best influence in vaccination was, and asked to select one answer, 247 (46.8%) women overall said their general practitioner (GP). Unfortunately, 100 answers were not given or spoiled due to multiple options selected so this was 247/428, 57.7% of all those who answered the question.

**Figure 3:**
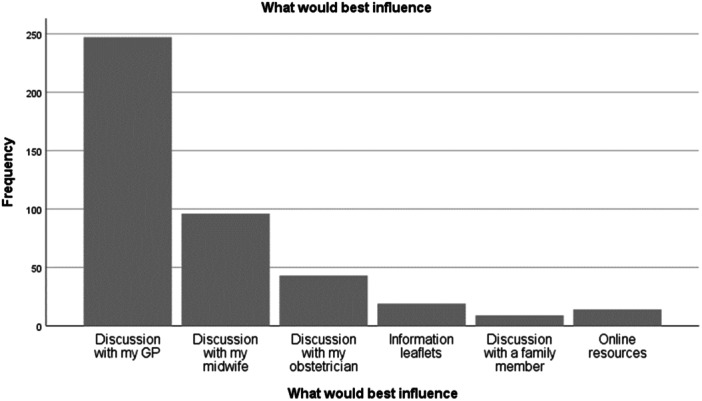
Influences or assistance in antenatal vaccination acceptance.

Smaller numbers preferred midwife, discussion with obstetrician, information leaflets, online resources, and consulting a family member, as shown in Figure 3.

## 4 DISCUSSION

This study is the largest survey to‐date of pregnant women on their awareness, and experience of RSV and attitudes to acceptance of antenatal RSV vaccination, when made available.

Comparing these results with Giles et al.,^13^ survey of 495 pregnant women in Australia where 83% had never heard of RSV, our level of awareness with 75.6% never hearing of RSV previously, appears comparable. Similarly, in the UK study by Wilcox et al.,^14^ 88% of women had no or little awareness of RSV, which is in line with our study showing 90.2% either had not heard of it or were unsure of its relevance in infants. Despite the low levels of awareness, 77% of women in the Australian study responded that they were very likely, and 75% of the women in the UK study that they were likely, to accept a vaccine if available,^13^ highlighting high rates of vaccine confidence. Among the study participants, 48.5% reporting that, they would avail of RSV vaccination and (45.8%) were unsure. However, the response of “no” to availing of the vaccine in our study was low at 5.3% and similar to the Australian responses for unlikely and very unlikely to accept at 5% combined. The participants of the Australian study rated proven safety as the most important information they would require before receiving the vaccine. Like our study, neither maternal age nor number of children had a statistically significant association with vaccine acceptance in that study.

According to the WHO, Healthcare workers remain the most trusted advisors and influencers in vaccination decisions,^12^ and this was again demonstrated in our study. The low levels of information seeking from online resources was an interesting finding in this highly digitalized world. Vaccination is frequently discussed online in mainstream and social media where the influence may be quite negative.^39^ Notably, it was also reported that the General Practitioner was the preferred source of information in a study by McQuaid et al.,^40^ on antenatal Group B Streptococcus (GBS vaccination which in itself was an online survey.

Examining vaccination in pregnancy generally, Hallissey et al. examined factors that influence uptake of vaccination in pregnancy in a group of 88 women recruited via general practices in Ireland. This study again showed healthcare provider recommendation as crucial to vaccine uptake and GPs as the most trusted source of information.^41^ Interesting findings in Wilcox's UK study which examined health professionals experience and views as well as pregnant women was that 66% of midwives had also never heard of RSV, and that 5% of healthcare professionals would be unlikely to support routine vaccination, though obstetricians were more likely to support initiatives than midwives.^14^ As such, significant education within the maternity and perinatal services would be essential to further support acceptance, especially given that midwives were the next most highly rated source of support for women in our study after GPs.

In general, vaccination prevents two to three million deaths per year worldwide and has scope to prevent a further 1.5 million with increased coverage.^11^ Low vaccination uptake, lack of vaccine knowledge (both in the general population and among healthcare professionals), attitudes towards vaccination including negative perceptions of efficacy and safety and a lack of confidence are all barriers to effective vaccination strategies across the life‐course.^11^ Specific barriers in the maternal immunization context, such as what was seen in our study, include low perceived risk of illness in the newborn infant and fear of harmful effects^42^ with concern regarding safety of vaccines in pregnancy cited as a barrier most frequently.^43^ The determinants of RSV vaccine acceptance, including vaccine education, communication strategies, and addressing of safety concerns were also echoed by Kherfan et al.^44^ in the recent paper validating a survey instrument on attitudes towards RSV vaccination. For maternal vaccination programs to be successful, pregnant women require awareness of the illness and its implications, robust information regarding vaccine safety and efficacy, endorsement by healthcare professionals and national professional bodies, and normalization of the practice as part of standard antenatal care.^43^, ^45^

With mathematical modeling studies on maternal vaccination predicting significant reduction of RSV‐ALRI related morbidity and mortality, especially among infants from LMICs,^46^ findings from this largest survey by the potential recipients, when candidate vaccines are in the pipeline can assist in shaping the public health and communication policies. In a June 2023 report, the Joint Committee on Vaccination and Immunization (JCVI) in England advocated for a universal program to protect newborns and infants from RSV, and subject to licensure of an appropriate product, including consideration of a maternal vaccine.^47^ Any “safety‐signals” identified during the trials of subunit maternal vaccines could be included in a comprehensive list for post‐approval monitoring as well, all with an aim to enhance the vaccine confidence among pregnant women.^48^

Willingness for RSV vaccination during pregnancy and an offer to vaccinate infants was recently studied in Italy during post‐COVID‐19 period with 490 participants, reporting higher preferred uptake for infants in comparison to pregnancy (61.1% vs. 45.9%).^49^ It is encouraging that women during pregnancy and lactational phase also endorsed the antenatal RSV vaccine acceptance as per another recent report from Kenya, highlighting the relatively high risk‐perception of RSV disease as a driver for vaccine readiness.^50^ In light of recent meta‐analysis suggesting maternal RSV vaccination offering effective antibody levels and diminishing RSV‐related severe disease among infants under 6 months of age, our survey results offers further impetus in the galvanization of antenatal vaccination as an integral component of a life‐course immunization approach.^51^

### 4.1 Limitations and strengths

We acknowledge the following limitations. (1) Recruitment was through a convenience sampling approach. All consecutive women could not be surveyed due to fluctuating clinical pressures and activity levels within the antenatal clinic study environment, and an opportunistic approach was also employed by the other two studies of this kind^13^, ^14^ (2) There is a potential risk‐of‐bias, as those least interested in antenatal vaccination may have been less likely to return the survey instrument, however, the response rate was monitored by sequential numbering of surveys. (3) There is potential selection bias within the overall study group as it is on pregnant women who have opted to register with the hospital and are actively seeking and attending antenatal care in this setting. (4) Additionally, it examines women who attended the public clinic only and not private consultants' rooms. (5) We did not stratify for parity, social class, weeks of gestation or ethnicity. (6) As COVID‐19 brought the topic of adult vaccination “to the dinner table” as never before, it is plausible that our survey instrument if repeated in the post‐COVID‐19 era (especially after the worldwide campaigns and uptake of maternal COVID‐19 vaccination), the RSV awareness and response towards maternal RSV vaccination could be different, and perhaps better.^52^

Despite these limitations, our survey results would be of value in the context of one or more maternal RSV vaccine approvals in the horizon. The response rate was high and thus largely representative of the population and generalizable to other settings. It has demonstrated low levels of RSV awareness but comparably high levels of interest in RSV vaccination with almost half of women stating that they would avail of the vaccine. A large proportion of women were undecided and so it was important to establish what would encourage uptake of the vaccine and where women would value support. Women highly rated their confidence in recommended vaccines and the protection of infants from illness as influencing their decision, and these factors were associated with increasing acceptance rates. Knowing sources for women to seek help in relation to decisions regarding antenatal vaccination allows targeted approaches and resource allocation where it will be of maximum benefit in reducing hesitancy, improving acceptance, and protecting infants.

## 5 CONCLUSION

Pregnant women demonstrated their willingness to accept antenatal RSV vaccination, with the protection of their infant from illness a key factor influencing their decisions. Women's preferences for advice regarding vaccination during pregnancy should be valued when planning antenatal vaccination programs given the strong desire for assistance in decision‐making from primary care setting. Key to the success of any strategies for the implementation of antenatal vaccine programs will be the education of all stakeholders‐ including policy‐makers, healthcare professionals and expectant mothers on the importance and safety of appropriate immunizations in the antenatal period.^13^ It is imperative that health workers are supported to provide trusted, credible information on vaccines.^11^ Interdisciplinary contact among professionals to include obstetricians, midwives, general practitioners, public health physicians, practice nurses, community nurses, neonatologists and pediatricians with a united approach will be necessary to overcome barriers and achieve maximum uptake. *Maternal immunization as a key pillar of life‐course vaccination strategy* would be important in the post‐COVID‐19 era and the findings of this survey would assist the targeted and focussed health education on RSV vaccination to pregnant women.

## AUTHOR CONTRIBUTIONS

**Siobhan McCormack**: Conceptualization; data curation; formal analysis; investigation; methodology; project administration; validation; visualization; writing—original draft; writing—review & editing. **Claire Thompson**: Conceptualization; data curation; methodology; project administration; writing—review & editing. **Miriam Nolan**: Data curation; project administration; writing—review & editing. **Mendinaro Imcha**: Data curation; methodology; project administration; writing—review & editing. **Anne Dee**: Investigation; methodology; supervision; writing—review & editing. **Jean Saunders**: Formal analysis; methodology; supervision; validation; writing—original draft; writing—review & editing. **Roy K Philip**: Conceptualization; data curation; formal analysis; investigation; methodology; project administration; supervision; validation; visualization; writing—review & editing.

## CONFLICT OF INTEREST STATEMENT

All authors completed the ICMJE forms and disclosed any conflicts. RKP received honorariums/travel expenses for conference presentations from Astra Zeneca® and Sanofi®. No conflict of interest for the other authors.

## ETHICS STATEMENT

Research ethics approval was granted by the University of Limerick Hospitals Group research ethics committee (REC Ref:101/18). Written informed consent was obtained from all participants in this study.

## Supporting information

Supporting information.

Supporting information.

## Data Availability

Data availability statement The anonymous data from this study is available on request from the authors.
