# Supplementary material for: Maternal awareness, acceptability and willingness towards respiratory syncytial virus (RSV) vaccination during pregnancy in Ireland
Source: Immun Inflamm Dis. 2024 Apr 25;12(4):e1257. doi: 10.1002/iid3.1257 (PMC11044221; doi:10.1002/iid3.1257)
Supplement: Supplementary file 1 — Supporting information. [file IID3-12-e1257-s001.docx]

***
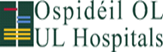

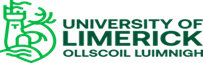
***

**Supplementary Information**

**Supplementary information S1 Survey questions**

| **Supplementary information S1 Survey questions** |
| --- |
| **Have you previously heard of Respiratory Syncytial Virus (RSV)?**   - Have not heard of it - Have some knowledge of it, but am unsure of its relevance in infants - Have some knowledge of it, including its significance in infancy - Have previous experience of it with my own child |
| **If a FULLY LICENCED and APPROVED maternal vaccine for use in pregnancy against RSV infection during infancy was made available, would you avail of it? Please select ONE answer**   - Yes - Don’t know - No |
| **What makes vaccination in pregnancy acceptable/not acceptable to you? Please select ALL that apply**   - I feel confident in recommended vaccines - I feel recommended vaccines will protect my baby from illness - I don’t think my baby is at risk of infection - I feel the vaccines could harm me - I feel the vaccines could harm my baby - I have no confidence in vaccines |
| **What would best influence or help in your decision regarding vaccination in pregnancy?**  **Please select ONE answer**   - Discussion with my GP - Discussion with my midwife - Discussion with my obstetrician - Information leaflets - Discussion with a family member - Online resources |
| **What is your Estimated Due Date?** |
