# Supplementary material for: Maternal awareness, acceptability and willingness towards respiratory syncytial virus (RSV) vaccination during pregnancy in Ireland
Source: Immun Inflamm Dis. 2024 Apr 25;12(4):e1257. doi: 10.1002/iid3.1257 (PMC11044221; doi:10.1002/iid3.1257)
Supplement: Supplementary file 2 — Supporting information. [file IID3-12-e1257-s002.docx]

***
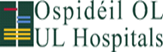

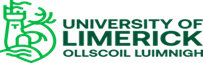
***

**Supplementary information S2 Information leaflet provided**

**Respiratory Syncytial virus (RSV)**

What is Respiratory syncytial virus (RSV)

RSV is the leading cause of viral lower respiratory tract infections in infants and young children.

RSV causes coughs and colds every winter and is the most common cause of bronchiolitis (inflammation of the small airways in the lung), a respiratory tract infection that commonly occurs in the first year of life.

How common is RSV?

There were over 2,500 cases of RSV reported in Ireland in winter 2016/2017. Globally, in 2015, RSV was responsible for 1.4 million admissions to hospital in infants under 6 months and 27,000 deaths in Infants under 6 months.

What is currently done against RSV?

No specific treatment other than treatment of symptoms is necessary for children with milder illness. Children with severe respiratory illness will require hospitalisation and oxygen therapy. Infants at high risk (for example extreme prematurity) require a costly monthly injection against RSV infection during the first winter season.

What is the consequence of RSV to the baby?

RSV is a significant cause of severe respiratory illness among children under 2 years of age and is also the most common cause of hospital admissions due to acute respiratory illness in young children. RSV infections are likely to be more severe in the first months of life than in older age groups. Many of the affected infants require high dependency and intensive care unit admission.

By two years of age, nearly all children have been infected with RSV at least once. Most cases are not specifically diagnosed as RSV; however, it causes 80% cases of bronchiolitis and 20% cases of pneumonia cases in young children. It is a significant cause of infection and outbreaks in hospitals, neonatal units and day units.

RSV Vaccine

No vaccines are currently available to prevent RSV infections. Vaccines against RSV has been identified as a priority by the World Health Organisation (WHO) and are currently in clinical trials in pregnant women.
